# DNA flow cytometric analysis in variable types of hydropic placentas

**Published:** 2015-05

**Authors:** Fatemeh Atabaki pasdar, Alireza Khooei, Alireza Fazel, Maryam Rastin, Nafise Tabasi, Tahmineh Peirouvi, Mahmoud Mahmoudi

**Affiliations:** 1 *Department of Anatomical Sciences, Urmia University of Medical Sciences, Urmia, Iran.*; 2 *Department of Pathology, Imam Reza Hospital, Mashhad University of Medical Sciences, Mashhad, Iran.*; 3 *Department of Anatomy and Cell Biology, Mashhad University of Medical Sciences, Mashhad, Iran.*; 4 *Immunology Research Center, BuAli Research Institute, Mashhad University of Medical Sciences, Mashhad, Iran.*

**Keywords:** *Flow cytometry*, *Hydatidiform mole*, *Spontaneous abortion*, *Ploidy*

## Abstract

**Background:**

Differential diagnosis between complete hydatidiform mole, partial hydatidiform mole and hydropic abortion, known as hydropic placentas is still a challenge for pathologists but it is very important for patient management.

**Objective:**

We analyzed the nuclear DNA content of various types of hydropic placentas by flowcytometry.

**Materials and Methods:**

DNA ploidy analysis was performed in 20 non-molar (hydropic and non-hydropic spontaneous abortions) and 20 molar (complete and partial moles), formalin-fixed, paraffin-embedded tissue samples by flow cytometry. The criteria for selection were based on the histopathologic diagnosis.

**Results:**

Of 10 cases histologically diagnosed as complete hydatiform mole, 9 cases yielded diploid histograms, and 1 case was tetraploid. Of 10 partial hydatidiform moles, 8 were triploid and 2 were diploid. All of 20 cases diagnosed as spontaneous abortions (hydropic and non-hydropic) yielded diploid histograms.

**Conclusion:**

These findings signify the importance of the combined use of conventional histology and ploidy analysis in the differential diagnosis of complete hydatidiform mole, partial hydatidiform mole and hydropic abortion.

## Introduction

Gestational trophoblastic disease (GTD) is a group of interrelated tumors originating from the placenta. Hydatidiform mole is the most common manifestation of GTD (1). It occurs in approximately 1 in every 1500 pregnancies in Europe and North America and is 3-10 times higher in Asian countries (2, 3). Previous studies demonstrated that women of Asian origin are at higher risk of developing moles than others (4). Hydatidiform moles are abnormal gestations characterized histologically by the presence of hydropic swelling affecting some or all of the chorionic villi accompanied by marked circumferential distribution of the villous trophoblast. It is usually benign but has malignant potentiality (1). 

Based on genetic and histopathologic features, hydatidiform mole can be subdivided into complete and partial mole. Placentas characterized by hydropic swelling of chorionic villi occur in a spectrum of pathologic conditions including hydropic abortion (HA), partial hydatidiform mole (PHM), and complete hydatidiform mole (CHM). Accurate diagnostic classification of hydropic placentas is important as the risk of persistent GTD is different among the 3 entities, Whereas HA is completely benign, hydatidiform moles carry a significant risk for developing persistent GTD, with the incidence of GTD being higher in patients with CHM (10-30%) than in patients with PHM (0.5-5%) (5, 6). 

Histologic examination forms the main tool in the diagnosis of molar pregnancies. However, there is considerable overlap in the histologic features between molar and nonmolar pregnancies and between CHMs and PHMs, resulting in significant interobserver variability in the diagnosis (7-9).

Cytogenetically, in most cases of CHMs, the chromosomal number is normal, 90% of cases have a 46 XX karyotype. The chromosomes are entirely of paternal origin due to fertilization of a nuclear egg by a haploid (23X) sperm which then duplicates its own chromosomes (10). The remaining 10% have a 46 XY karyotype, where all chromosomes are of paternal origin and result from dispermy (11). In a minority of cases, the DNA pattern is tetraploid (12). In contrast, partial hydatidiform moles are almost always triploid (69XXX or 69XXY), with the extra haploid set of chromosomes derived from the father and a few show trisomy 16 (12-14). Spontaneous abortions are usually diploid; triploidy is thought to occur in approximately 8-11% of all spontaneous abortions (15-19). Pathologists now rely on molecular techniques that make use of DNA content and origin differences; however most of these techniques must be applied to living cells, which is seldom available. Flowcytometry has become widely accepted as a reliable test for ploidy which analyses a large number (10000- 20000) of random nuclei (20, 21). Moreover, it can be applied to cases embedded in paraffin.

The aim of this study was to evaluate the results of DNA flowcytometry in various types of hydropic placentas.

## Materials and methods

**Case selection**

In this descriptive retrospective study, formalin-fixed, paraffin-embedded gestational products from 40 placental tissue samples, including 10 CHMs, 10 PHMs, 10 hydropic (HA) and 10 non-hydropic or simple spontaneous abortions (SA) were retrieved from the files of the Department of pathology, Imam Reza and Qaem Hospitals, Mashhad University of Medical Sciences, Mashhad, Iran, since April 2007 to April 2011. All samples were taken from women with gestational age between 11-12 weeks. Tissue sections of the specimens were stained with routine hematoxylin-eosin and histopathologically reviewed for tissue adequacy and confirmation of diagnosis. Diagnoses were made by surgical pathologists using published criteria (12). 

Namely the diagnosis of a CHM was made when there was complete hydatidiform change from edema to central cisterna formation, absence of an embryo and conspicuous trophoblastic hyperplasia. The diagnosis of a PHM was made when there was partial villous involvement (normal and edematous villi), the presence of an embryo or fetus, mild to moderate focal trophoblastic hyperplasia and trophoblastic inclusion. Trophoblastic hyperplasia is an essential feature in differentiating PHMs from hydropic and non-hydropic abortions. The samples with inadequate or necrotic tissues were excluded.

**Flow cytometry**

Flow cytometric DNA analysis was performed on formalin-fixed, paraffin- embedded tissue blocks. The selection criterion for the blocks was the presence of both placental and maternal (decidual) tissue in approximately such amounts that representative DNA histograms could be anticipated. 

Maternal tissue had to be present as the internal diploid control. One 50 μm section of each block was placed in 10 ml glass centrifuge tubes and dew axed using two changes of xylene, 3 ml for 10 min at room temperature, and then rehydrated in a sequence of 3 ml of 100%, 95%, 75%, and 50% ethanol for 10 min each at room temperature with centrifugation and decantation of the supernatant after each step. 

The tissues was then washed twice in distilled water and resuspended in pepsin solution (1 ml of 0.05% pepsin in 0.9% NaCl, pH 1.5) at 37^o^C for 45-60 min with intermittent mixing using a vortex. The reaction was stopped with cold PBS and the samples were washed twice with phosphate buffered saline (PBS). 

The resulting cell suspension was washed twice with PBS. After addition of RNase to remove any nuclear or residual cytoplasmic RNA, and propidium iodide, ploidy was determined by flowcytometry using FACS Calibur flowcytometer (Becton-Dickinson). Histograms were generated from analysis of 10000 nuclei and displayed as linear fluorescence.

As the use of internal standard controls, the first peak in the histograms was considered to represent diploid cells. When two distinct peaks were present, the DNA index (DI) was calculated by dividing the modal channel number of the peak with higher DNA content by that of the peak with lower DNA content, if DI value being between 1.4 and 1.6 it was classified as triploid, and it was considered as tetraploid if the peak in the G_2_/M region represented greater than 25% of the cells and the DI was between 1.90 and 2.10.

**Statistical analysis**

Coefficients of variation (CV) were assessed with the use of the computer program Lysys II Software (Becton-Dickinson, Mountain View, CA, USA).

## Results

Interpretable DNA histograms were obtained from all samples. The results of DNA ploidy are summarized in Table I. Of 10 cases histologically diagnosed as complete hydatiform mole, 9 cases yielded diploid histograms, and 1 case was tetraploid. Of 10 partial hydatidiform moles, 8 were triploid and 2 were diploid. All of 20 cases diagnosed as spontaneous abortions (hydropic and non- hydropic) were diploid. The average coefficient of variation for the G0/G1 peak was 7.71% (4.06-24.64%).

**Table I:** DNA ploidy in hydatidiform moles and abortions using flow cytometric analysis

**Histologic diagnosis**	**DNA-ploidy pattern**		
	**Diploid **	**Triploid **	**Tetraploid **
Complete hydatidiform moles	9 (90)	**-**	1 (10)
Partial hydatidiform moles	2 (20)	8 (80)	**-**
Hydropic abortion	10 (100)	**-**	**-**
Non-hydropic abortion	10 (100)	**-**	**-**

Data are presented as n (%).

**Figure 1:**
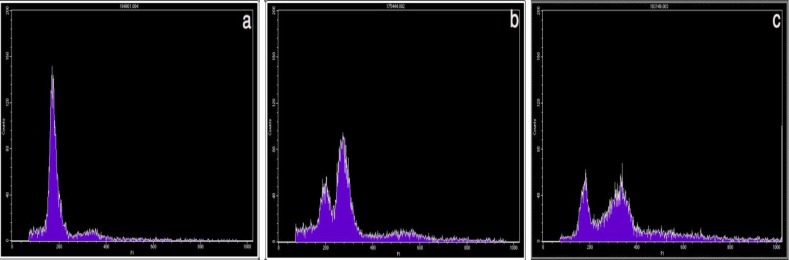
Examples of the three kinds of DNA histograms. Vertical axis, number of counted events; horizontal axis, channel number, representing the relative DNA content. (A) Normal diploid DNA histogram. One high peak is considered to be diploid maternal and placental cell populations. The small peak represents the G_2_/M cells. (B) DNA histogram expressing triploidy. The first peak represents maternal diploid cells and the second peak represents placental cells with a triploid DNA content. (C) DNA histogram expressing tetraploidy. The first peak represents maternal diploid cells and the second peak represents placental cells with a tetraploid DNA content

## Discussion

The differentiation of complete mole from partial mole and hydropic abortion is very important for patient management. Most histology-based diagnostic criteria define classic features seen in well-formed moles (22, 23). Increasing use of prenatal β-hCG monitoring and high-resolution ultrasound now permits earlier clinical recognition of abnormal pregnancies (24). Molar pregnancies are being evacuated early in gestation, before the 

development of well-established classic morphologic features, thus adding to the difficulty in diagnosis. In the studies assessing the intra- and inter-observer agreement among a group of pathologists in diagnosis of molar pregnancies, Howat *et al* and Fukunaga *et al* found that complete mole could reliably distinguished from non-molar pregnancy, but neither non-molar pregnancy nor complete mole could be easily differentiated from partial mole (8, 9).

Considering the risk of molar pregnancies to developing persistent gestational trophoblastic tumors, most of authors have emphasized the importance of some ancillary tools as cytometry and histochemistry to improve differential diagnosis of hydropic placentas (19, 21, 25, 26). In this study 9 of 10 cases histologically diagnosed as CHMs, yielded diploid histograms by flowcytometry. A tetraploid pattern was seen in the remaining case. No significant histologic difference was found between the tetraploid and diploid CHMs. Fukunaga found that of 35 specimens of formalin-fixed, paraffin-embedded, tetraploid hydropic villous tissues, 25 were CHMs, 10 were HAs and none were partial moles (27). Osterheld reported that tetraploid CHMs occur in older patients (mean: 30.4 years; range: 27-36 years) compared to the patients with diploid moles (mean: 27.3 years; range: 19-31 years) (25). 

Another study done by Fukunaga *et al* showed that of 239 complete moles, there were 182 diploid, 30 tetraploid and 27 aneuploidy cases. Furthermore, they reported that their results suggest that aneuploidy CHMs are associated with less risk for persistent disease than diploid or tetraploid CHMs (28). In the majority of PHMs, a DNA-triploid pattern was found. 2 of 10 cases, histologically diagnosed as PHMs were diploid. A few diploid PHMs have been described, although it has been suggested that diploid PHMs probably do not exist, with most reported cases being misdiagnosed CHMs (29). Furthermore, the pattern of trophoblastic hyperplasia which was multi focal or cicumferrential in both cases refuse the possibility of HAs which have polar trophoblastic proliferation (30). 

These data suggesting a possible wrong orientation of the histological diagnosis (PHM instead CHM). In cases of discordance between the histologic diagnosis and the results of flowcytometry, reexamination of the histologic specimens is required (28). In these 2 discordant cases, the original hematoxylin-eosin stained sections were reviewed with knowledge of the ploidy status. In both cases, the histological diagnosis was revised to CHM. One ploidy analysis study performed by Crisp *et al* showed that 13/16 cases, histologically diagnosed as partial moles, were demonstrated to be triploid, the remaining three cases were diploid. The discordant cases were reviewed with knowledge of the ploidy and P57 immunohistochemistry status and accordingly these cases were reclassified as non-molar pregnancies (31). 

All of the HAs and SAs yielded diploid histograms. It must be noted that among karyotypic abnormalities, flow cytometric analysis on paraffin-embedded material can detect only polyploidies. Trisomies, monosomies and structural anomalies cannot be detected (32). The most frequent type of chromosomal abnormalities, detected in spontaneous abortions were autosomal trisomies, though these diploid histograms might have been trisomic abortions, which cannot be assessed by DNA flowcytometry (17, 19). 

In summary, no single technique can be used to make the diagnosis of hydatidiform moles; ploidy is only of value once the diagnosis of hydatidiform mole has been made histologically, as diploid placental tissue may have originated from a complete mole or a hydropic miscarriage.

## Conclusion

These findings signify the importance of the combined use of conventional histology and ploidy analysis in the differential diagnosis of complete hydatidiform mole, partial hydatidiform mole and hydropic abortion.

## Note

***This article extracted from Ph.D thesis (Fatemeh Atabaki Pasdar).***

## References

[1] Soper JT, Mutch DG, Schink JC, American College of Obstetricians Gynecologists (2004). Diagnosis and treatment of gestational trophoblastic disease. Gynecol Oncol.

[2] Bracken MB (1987). Incidence and etiology of hydatidiform mole: an epidemiological review. Br J Obstet Gynaecol.

[3] Steigrad SJ (2003). Epidemiology of gestational trophoblastic diseases. Best Pract Res Clin Obstet Gynecol.

[4] Tham BW, Everard JE, Tidy JA, Drew D, Hancock BW (2003). Gestational trophoblastic disease in the Asian population of Northern England and North Wales. Br J Obstet Gynaecol.

[5] Berkowitz RS, Goldstein DP (2009). Current management of gestational trophoblastic diseases. Gynecol Oncol.

[6] Scoper JT, Lewis Jr, Hammond CB, Hoskins WJ, Perez CA, Young RC (1997). Gestational trophoblastic disease. Principles and practice of gynecologic oncology.

[7] Messerli ML, Parmley T, Woodruff JD, Lilienfeld AM, Bevilacqua L, Rosenshein NB (1987). Inter- and intra-pathologist variability in the diagnosis of gestational trophoblastic neoplasia. Obstet Gynecol.

[8] Howat AJ, Beck S, Fox H, Harris SC, Hill AS, Nicholson CM (1993). Can Histopathologists Reliably Diagnose Molar Malignancy?. J Clin Pathol.

[9] Fukunaga M, Katabuchi H, Nagasaka T, Mikami Y, Minamiguchi S, Lage JM (2005). Interobserver and intraobserver variability in the diagnosis of hydatidiform mole. Am J Surg Pathol.

[10] Kajii T, Ohama K (1977). Androgenetic origin of hydatidiform mole. Nature.

[11] Pattillo RA, Sasaki S, Katayama KP, Roesler M, Mattingly RF (1981). Genesis of 46,XY hydatidiform mole. Am J Obstet Gynecol.

[12] Rosai J, Ackerman S (2011). Female reproductive system-placenta. surgical pathology.

[13] Lawler SD, Fisher RA, Dent J (1991). A prospective genetic study of complete and partial hydatidiform moles. Am J Obstet Gynecol.

[14] Lage JM, Mark SD, Roberts DJ, Goldstein DP, Bernstein MR, Berkowitz RS (1992). A flow cytometric study of 137 fresh hydropic placentas: correlation between types of hydatidiform moles and nuclear DNA ploidy. Obstet Gynecol.

[15] Bentley RC (2003). Pathology of gestational trophoblastic disease. Clin Obstet Gynecol.

[16] Watanabe M, Ghazizadeh M, Konishi H, Araki T (1998). Interphase cytogenetic and AgNOR analysis of hydatidiform moles. J Clin Pathol.

[17] Cunningham F, Leveno K, Bloom S, Hauth J, Rouse D, Spong C (2010). Spontaneous abortion. Williams Obstetrics.

[18] Fukunaga M, Ushigome S, Fukunaga M, Sugishita M (1993). Application of cytometry in diagnosis of hydatidiform moles. Mod Pathol.

[19] Van Oven MW, Schoots CJF, Oosterhunis JW, Keij JF, Dam-Meiring A, Huisjes HF (1989). The use of DNA flow cytometry in the human abortions. Hum Pathol.

[20] Hemming JD, Quirke P, Womack C, Wells M, Elston CW, Bird CC (1987). Diagnosis of molar pregnancy and persistent trophoblastic disease by flow cytometry. JClin Pathol.

[21] Lage JM, Bagg A (1996). Hydatidiform moles: DNA flow cytometry, image analysis and selected topics in molecular biology. Histopathology.

[22] Szulman AE, Surti U (1978). The syndromes of hydatidiform mole I. Cytogenetic and morphologic correlations. Am J Obstet Gynecol.

[23] Paradinas FJ, Browne P, Fisher RA, Foskett M, Bagshawe KD, Newlands E (1996). A clinical, histopathological and flow cytometric study of 149 complete moles, 146 partial moles and 107 non-molar hydropic abortions. Histopathology.

[24] Hancock BW, Tidy JA (2002). Current management of molar pregnancy. J Reprod Med.

[25] Osterheld MC, Caron L, Chaubert P, Meagher-Villemure K (2008). Combination of immunohistochemistry and ploidy analysis to assist histopathological diagnosis of molar diseases. Clin Med Pathol.

[26] Atabaki F, Khooei AR, Fazel AR, Mahmoudi M, Nikravesh MR, Khaje Delui M (2012). Diagnostic value of lectins in differentiation of molar placentas. IJBMS.

[27] Fukunaga M, Endo Y, Ushigome S (1996). Clinicopathologic study of tetraploid hydropic villous tissues. Arch Pathol Lab Med.

[28] Fukunaga M (2001). Flow cytometric and clinicopathologic study of complete hydatidiform moles with special refrence to the significance of cytometric aneuploidy. Gynecol Oncol.

[29] Genest DR, Ruiz RE, Weremowicz S, Berkowitz RS, Goldstein DP, Dorfman DM (2002). Do nontriploid partial hydatidiform moles exist? A histologic and flow cytometric reevaluation of nontriploid specimens. J Reprod Med.

[30] Nga A, Cheung Y (2003). Patholgy of gestationl trophoblastic diseases. Best Pract Res Clin Obstet Gynaecol.

[31] Crips H, Burton JL, Stewart R, Wells M (2003). Refining the diagnosis of hydatidiform mole: image ploidy analysis and p57KIP2 immunohistochemistry. Histopathology.

[32] Cera G, Fruttero A, Rua S, Comino A, Abrate M (1992). Flow cytometric studies in spontaneous abortions. Applications in the medico-legal practice. Forensic Sci Int.

